# Pseudologia Fantastica: A Systematic Review of Sociodemographic Features and Psychiatric Comorbidities

**DOI:** 10.1192/j.eurpsy.2026.11904

**Published:** 2026-07-31

**Authors:** V. Sharma, R. K. Suri, T. Kainth, M. Salehi, G. Gill, K. Tran, S. Gunturu

**Affiliations:** 1 Dayanand Medical College & Hospital, Ludhiana, India; 2 Psychiatry, BronxCare Health System, The Bronx; 3 Psychiatry, Johns Hopkins University School of Medicine, Baltimore; 4 Psychiatry, Case Western Reserve University/University Hospitals, Cleveland, United States

## Abstract

**Introduction:**

Pseudologia fantastica (PF), also referred to as pathological lying in literature, consists of fantastically fabricated distortions with no apparent reason or personal gain. PF is not recognized as an independent clinical entity in the DSM-V, resulting in a diagnostic challenge and significant legal implications for competency assessment to stand trial.

**Objectives:**

Discuss demographics, intention and content of the fabrications, reaction on confrontation, psychiatric comorbidities and concurrent substance abuse associated with pseudologia fantastica through a systematic review and qualitative analysis.

**Methods:**

We conducted a systematic search through 3 databases - PubMed, PsycINFO, and Scopus, to discuss the demographics, substance use, dynamics of the lies told by the patients with PF, and their psychiatric comorbidities. After applying inclusion and exclusion criteria for the title, abstract, and full-text assessments, we included a total of 34 articles consisting of 55 cases of PF in our final study. PRISMA guidelines were followed throughout (Figure 1).

**Results:**

A total of 55 cases from existing literature were selected and evaluated. Approximately 49% of pseudologues presented in their 3rd and 4th decade of life with a marked male predominance (72.73%). The three leading motives behind the fabrications seemed to be self-aggrandizing (38.18%), seeking attention (36.36%), and assumption of a sick role (29.09%). Exaggerations about life events (38.18%), education, and occupation (43.64%) closely followed health-related lies (67.27%) closely (Figure 2). Pseudologues assumed a different name/identity (32.73%), which posed serious legal ramifications. The most common psychiatric comorbidities associated with PF were cluster B personality disorders (25.45%), depressive disorders (16.36%), trauma/stress disorders (16.36%), and somatic and factitious disorders (14.55%) (Figure 3). Pseudologues also exhibited high suicidality (27.27%), with 16.36% attempting suicide. Substance abuse was also prevalent (30.91%), with significant alcohol use disorder (21.82%).

**Image 1:**
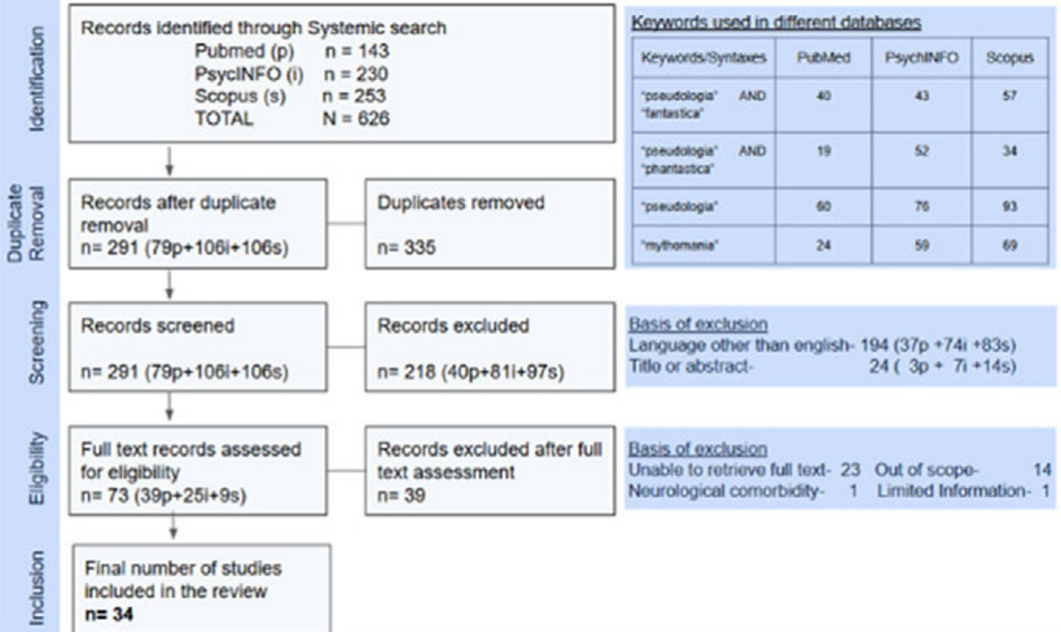
Image 1: Long description.

**Image 2:**
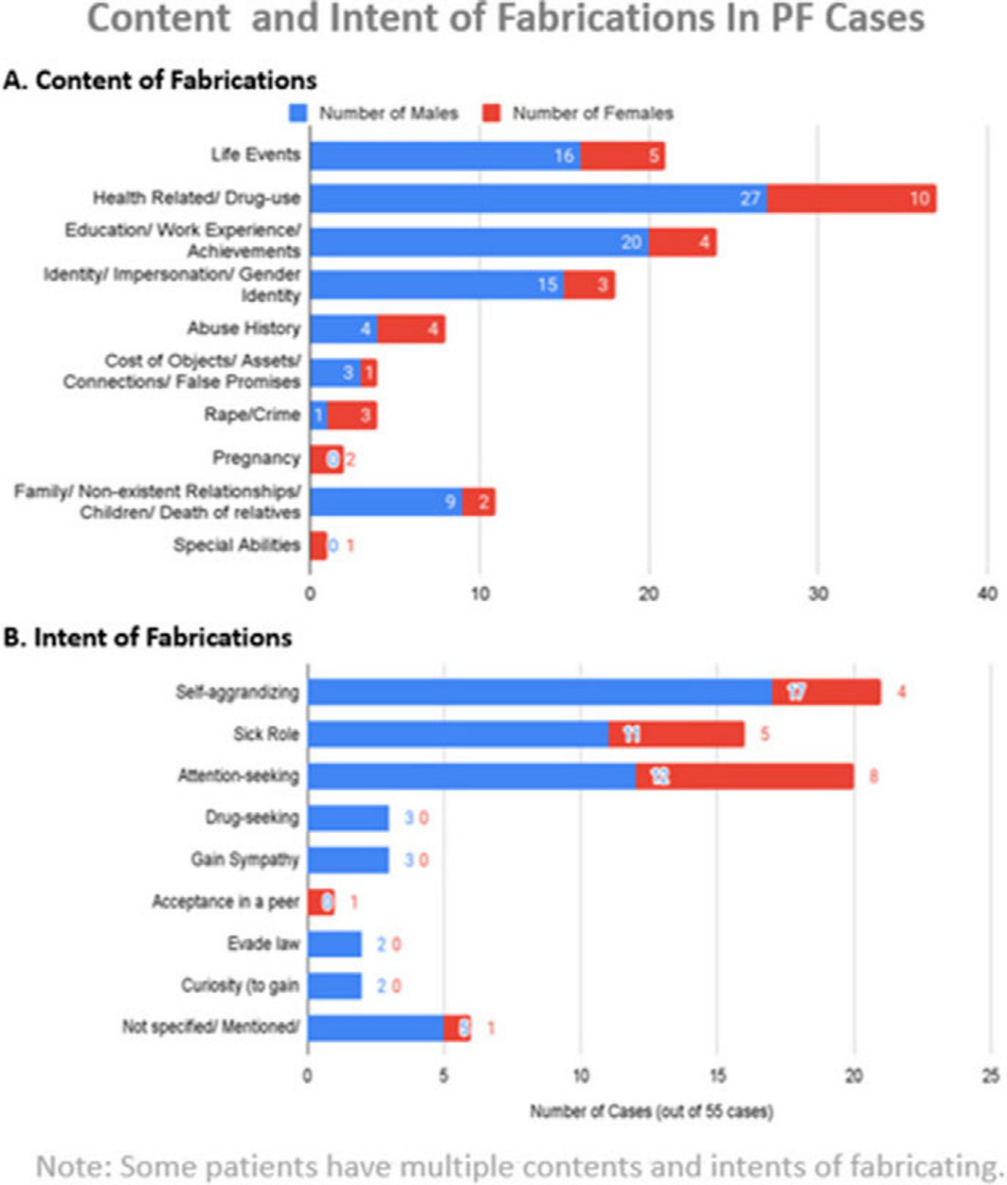
Image 2: Long description.

**Image 3:**
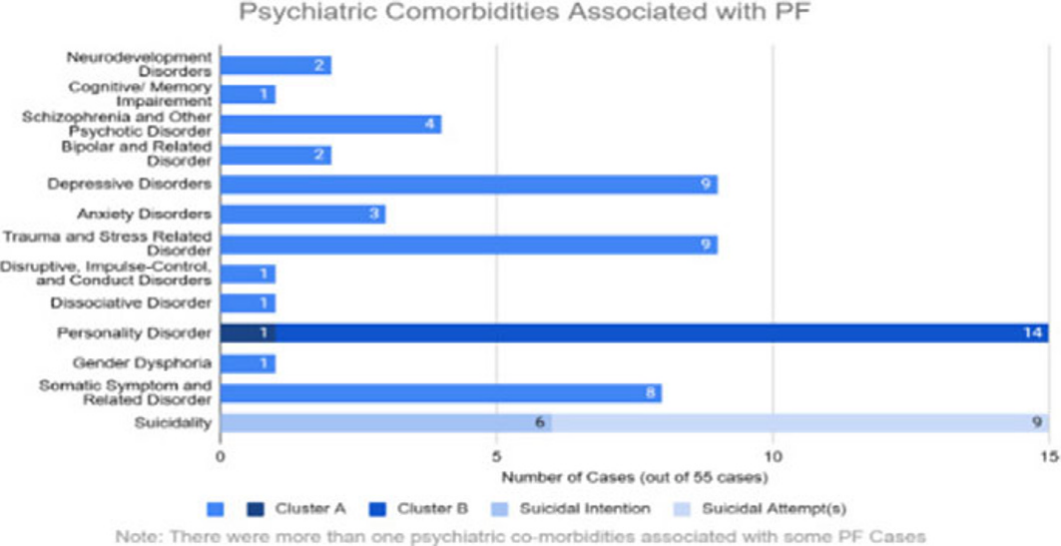
Image 3: Long description.

**Conclusions:**

PF puts considerable strain on employment, societal relationships, and legal and medical systems, and it is crucial to identify it early and empower physicians and law practitioners on best practices to manage the condition.

**Disclosure of Interest:**

None Declared

## Long descriptions

### Image 1: Long description

The flowchart is divided into five vertical stages.

1. Identification. A top box lists records from Pubmed p n equals 143, Psych I N F O i n equals 230, and Scopus s n equals 253, totaling N equals 626. To the right, a table shows keyword hits for pseudologia fantastica, pseudologia phantastica, pseudologia, and mythomania across the three databases.

2. Duplicate Removal. 335 duplicates were removed, leaving n equals 291 records. This is broken down as 79 p plus 106 i plus 106 s.

3. Screening. 291 records were screened and 218 were excluded. A list to the right specifies exclusion reasons. Language other than English accounted for 194 37 p plus 74 i plus 83 s. Title or abstract accounted for 24 3 p plus 7 i plus 14 s.

4. Eligibility. 73 full text records 39 p plus 25 i plus 9 s were assessed. 39 were excluded. Reasons listed to the right include. Unable to retrieve full text 23. Out of scope 14. Neurological comorbidity 1. Limited Information 1.

5. Inclusion. The final box at the bottom indicates the final number of studies included in the review is n equals 34.

Navigate back to Image 1:.

### Image 2: Long description

Panel A, Content of Fabrications, uses an x-axis from 0 to 40. Blue bars represent Number of Males and red bars represent Number of Females.

* Life Events: 16 males, 5 females.

* Health Related/ Drug-use: 27 males, 10 females.

* Education/ Work Experience/ Achievements: 20 males, 4 females.

* Identity/ Impersonation/ Gender Identity: 15 males, 3 females.

* Abuse History: 4 males, 4 females.

* Cost of Objects/ Assets/ Connections/ False Promises: 3 males, 1 female.

* Rape/ Crime: 1 male, 3 females.

* Pregnancy: 0 males, 2 females.

* Family/ Non-existent Relationships/ Children/ Death of relatives: 9 males, 2 females.

* Special Abilities: 0 males, 1 female.

Panel B, Intent of Fabrications, uses an x-axis from 0 to 25 representing Number of Cases out of 55 cases.

* Self-aggrandizing: 17 males, 4 females.

* Sick Role: 11 males, 5 females.

* Attention-seeking: 12 males, 8 females.

* Drug-seeking: 3 males, 0 females.

* Gain Sympathy: 3 males, 0 females.

* Acceptance in a peer: 0 males, 1 female.

* Evade law: 2 males, 0 females.

* Curiosity to gain: 2 males, 0 females.

* Not specified/ Mentioned: 5 males, 1 female.

A note at the bottom states that some patients have multiple contents and intents of fabricating.

Navigate back to Image 2:.

### Image 3: Long description

The horizontal bar chart displays the number of cases for 13 psychiatric categories associated with P F. The X axis represents the Number of Cases out of 55 cases, ranging from 0 to 15.

From top to bottom, the data points are:

* Neurodevelopmental Disorders: 2 cases.

* Cognitive/Memory Impairment: 1 case.

* Schizophrenia and Other Psychotic Disorder: 4 cases.

* Bipolar and Related Disorder: 2 cases.

* Depressive Disorders: 9 cases.

* Anxiety Disorders: 3 cases.

* Trauma and Stress Related Disorder: 9 cases.

* Disruptive, Impulse-Control, and Conduct Disorders: 1 case.

* Dissociative Disorder: 1 case.

* Personality Disorder: This bar is divided into Cluster A (1 case) and Cluster B (14 cases), totaling 15.

* Gender Dysphoria: 1 case.

* Somatic Symptom and Related Disorder: 8 cases.

* Suicidality: This bar is divided into Suicidal Intention (6 cases) and Suicidal Attempt(s) (9 cases), totaling 15.

A legend at the bottom identifies the color coding for Cluster A, Cluster B, Suicidal Intention, and Suicidal Attempt(s). A note states that there were more than one psychiatric co-morbidity associated with some P F cases.

Navigate back to Image 3:.

